# Semaphorin 3A Is a New Early Diagnostic Biomarker of Experimental and Pediatric Acute Kidney Injury

**DOI:** 10.1371/journal.pone.0058446

**Published:** 2013-03-04

**Authors:** Calpurnia Jayakumar, Punithavathi Ranganathan, Prasad Devarajan, Catherine D. Krawczeski, Stephen Looney, Ganesan Ramesh

**Affiliations:** 1 Department of Medicine and Vascular Biology Center, Georgia Health Sciences University, Augusta, Georgia, United States of America; 2 Department of Nephrology and Hypertension, Cincinnati Children’s Hospital Medical Center, University of Cincinnati School of Medicine, Cincinnati, Ohio, United States of America; 3 Heart Institute, Cincinnati Children’s Hospital Medical Center, University of Cincinnati School of Medicine, Cincinnati, Ohio, United States of America; 4 Department of Biostatistics, Georgia Health Sciences University, Augusta, Georgia, United States of America; University of Sao Paulo Medical School, Brazil

## Abstract

**Background:**

Semaphorin 3A is a secreted protein that regulates cell motility and attachment in axon guidance, vascular growth, immune cell regulation and tumor progression. However, nothing is known about its role in kidney pathophysiology. Here, we determined whether semaphorin3A is induced after acute kidney injury (AKI) and whether urinary semaphorin 3A can predict AKI in humans undergoing cardiopulmonary bypass (CPB).

**Methods and Principal Findings:**

In animals, semaphorin 3A is localized in distal tubules of the kidney and excretion increased within 3 hr after reperfusion of the kidney whereas serum creatinine was significantly raised at 24 hr. In humans, using serum creatinine, AKI was detected on average only 48 hours after CPB. In contrast, urine semaphorin increased at 2 hours after CPB, peaked at 6 hours (2596±591 pg/mg creatinine), and was no longer significantly elevated 12 hours after CPB. The predictive power of semaphorin 3A as demonstrated by area under the receiver-operating characteristic curve for diagnosis of AKI at 2, 6, and 12 hours after CPB was 0.88, 0.81, and 0.74, respectively. The 2-hour urine semaphorin measurement strongly correlated with duration and severity of AKI, as well as length of hospital stay. Adjusting for CPB time and gender, the 2-hour semaphorin remained an independent predictor of AKI, with an odds ratio of 2.19.

**Conclusion:**

Our results suggest that semaphorin 3A is an early, predictive biomarker in experimental and pediatric AKI, and may allow for the reliable early diagnosis and prognosis of AKI after CPB, much before the rise in serum creatinine.

## Introduction

Acute kidney injury (AKI) due to ischemia is a frequent and serious complication in the hospital setting. The incidence of AKI is increasing world-wide, affecting about 6% of all hospitalized patients in whom it is an independent predictor of mortality and morbidity [1]–[3]. In the critical care setting, the prevalence of AKI requiring dialysis is about 6%, with a mortality rate exceeding 60% [4]. Once established, the treatment is largely supportive, at an annual cost surpassing $10 billion in the US alone (5). The diagnosis currently depends on detection of reduced kidney function by the rise in serum creatinine concentration, which is a delayed and unreliable measure in the acute setting [5]. Ironically, experimental studies have identified interventions that may prevent or treat AKI if instituted early in the disease process, well before the serum creatinine rises [6]. The lack of early predictive biomarkers has impaired our ability to translate these promising findings to human AKI.

Cardiopulmonary bypass (CPB) surgery is the most frequent major surgical procedure performed in hospitals worldwide, with well over a million operations undertaken each year. Acute kidney injury (AKI) is a frequent and serious complication encountered in 30–40% of adults and children after CPB [7]–[9]. AKI requiring dialysis occurs in up to 5% of these cases, in whom the mortality rate approaches 80%, and is indeed the strongest independent risk factor for death [10]. However, even a minor degree of post-operative AKI as manifest by only a 0.2–0.3 mg/dl rise in serum creatinine from baseline is also associated with a significant increase in mortality [11]; [12]. Additionally, AKI after cardiac surgery is associated with adverse outcomes such as prolonged intensive care and hospital stay, dialysis dependency, and increased long-term mortality [13]. Infants and children with congenital heart diseases may be especially vulnerable to developing AKI, since many require multiple surgeries for step-by-step repair of complex congenital anomalies[14]–[16]. These patients comprise an important population for the initial validation of AKI biomarkers, since they do not exhibit common co-morbid variables that complicate similar studies in adults, such as diabetes, hypertension, atherosclerosis, and nephrotoxin use [17].

Experimental studies aimed at a better understanding of the early adaptive response of the stressed kidney have recently yielded several candidate genes and proteins that are serendipitously emerging as non-invasive candidate biomarkers of AKI [8]; [9]; [18]; [19]. Here we describe the identification and validation of a new early diagnostic biomarker, semaphorin 3A, for acute kidney injury. The Semaphorins make up the largest family of axon guidance cues yet described. Semaphorins are divided into 8 classes (classes 3–7 found in vertebrates). Class 3 Semaphorins are secreted, classes 4 through 6 are transmembrane proteins, and class 7 are membrane associated via glycosylphosphatidylinositol (GPI) linkage. They are characterized structurally by a conserved ∼400 amino acid sema domain [20]. They are classically described as collapsing factors and mediators of axon repulsion, although they may also act as context-dependent chemoattractants. Semaphorins have been shown to have roles in cardiovascular development and in the regulation of immune cell antigen presentation. In addition semaphorins are also known to regulate cell motility and attachment in axon guidance, vascular growth, immune cell regulation and tumor progression [20]–[23]. Semaphorin3A is a chemorepellent with multiple guidance functions, including axon pathfinding, cardiac and peripheral vascular patterning and branching morphogenesis. Semaphorin 3A gene deletion results in perinatal lethality [24]. Semaphorin3A signaling is mediated by a complex of the binding receptor neuropilin 1 and the signaling receptors plexinA1 or A3 [25]; [26]. Both Semaphorin3A and its receptor neuropilin 1 are expressed in the developing glomerulus, and Semaphorin3A remains expressed in adult podocytes and collecting tubules [27]; [28]. Semaphorin3A inhibits ureteric bud branching by downregulation of glial cell-line-derived neurotrophic factor [23]. The regulation of Semaphorin3A expression in kidney and pathophysiological role is unknown. Therefore, the objective of this study was to determine the expression pattern in animal models of AKI, its functional role in AKI, and whether urinary semaphorin 3A levels predict the development of AKI in animal and human acute kidney injury.

## Materials and Methods

### Ethics Statement

All animal handling and procedures were performed in accordance with protocols for these studies that have been approved by the Institutional Animal Care and Use Committee at the Georgia Health Sciences University (Approval # BR10-10-369). All surgery was performed aseptically under deep anesthesia, and every attempt was made to minimize pain and discomfort. Human studies were approved by Human Assurance Committee at Georgia Health Sciences University and the institutional review board of the Cincinnati Children's Hospital Medical Center. Written informed consent was obtained from the legal guardian of every child before enrolment. Written informed consent was approved by the institutional review board of the Cincinnati Children's Hospital Medical Center.

#### Animal model of acute kidney injury

8–10 weeks old male C57BL/6J mice was in our studies (The Jackson Laboratory, Bar Harbor, Maine, USA). Ischemia reperfusion induced acute kidney injury or sham operation was carried out as described before [29]; [30]. Urine samples were collected at 3, 6, 24, 48 hr after reperfusion. Cisplatin (MP Biomedicals) was administered at a dose of 25 mg/kg BW, intraperitoneally and animals were sacrificed at 72 hr for collection of kidney tissue. Diabetes was induced by administering streptozotocin (STZ) (SIGMA-ALDRICH., Co.) (100 mg/kg BW). 3 weeks after diabetes urine samples were collected and sacrificed to harvest kidney tissues.

### Immunohistochemical Analysis

Kidneys were fixed in 10% buffered formalin overnight at room temperature, transferred to 70% ethanol for 24 h, and paraffin embedded. The kidneys were sectioned at a thickness of 4 µm onto Superfrost plus slides and processed. Slides were incubated in the absence or presence of primary antibodies to semaphorin 3A (Abcam) in humidified chambers overnight at 4°C, followed by incubation with biotin-conjugated donkey anti-rabbit IgG (Jackson ImmunoResearch Laboratories, West Grove, PA) for 1 hr at room temperature. Color was developed after incubation with ABC reagent (Vector Lab). The stained sections were photographed with an Olympus BX40 microscope (Olympus America, Melville, NY) on bright-field setting fitted with a digital camera (Olympus DP12; Olympus America) (magnification x660).

#### Analysis of semaphorin 3A by western blot analysis

A volume of urine containing 1 µg of creatinine for each mouse sample and 15 µg creatinine for each human sample was subjected to Western blot analysis of semaphorin 3A. Urine sample was loaded onto 4–12% polyacrylamide gels, separated, and then transferred onto a PVDF membrane. The membrane was probed with rabbit anti-semaphorin antibody (Abcam) or goat anti-semaphorin 3A antibody (Santacruz Biotechlogy). Proteins were detected using enhanced chemiluminescence detection reagents (Amersham Pharmacia Biotech, Inc.). To determine whether proteases in urine cleave intact recombinant semaphorin 3A, 250 ng of semaphorin 3A was incubated in 10 µl human urine for 1 hr at 37°C. some samples were treated with 20 mM EDTA. At the end of incubation, samples were loaded onto 4–12% polyacrylamide gels, separated, and then transferred onto a PVDF membrane. The membrane was probed with rabbit anti-semaphorin 3A antibody from Abcam (Cat # ab23393) and Santacruz Biotechnology (Cat # SC-1148).

#### Quantification of mouse semaphorin 3A in serum and urine

Mouse semaphorin 3A was quantified using an ELISA kit (Cat no: CSB-EL020980MO, Cedarlane Laboratories USA).

#### Renal function

Renal function was assessed by measurements of serum creatinine (cat no: DZ072B, Diazyme Labs, USA).

#### Quantification of mRNA by real-time RT-PCR

RNA was isolated using TRIZOL reagent (Life Technologies, Grand Island, NY). Real-time RT-PCR was performed in an Applied Biosystems Inc. 7700 Sequence Detection System (Foster City, California, USA). 3 µg total RNA was reverse transcribed in a reaction volume of 40 µl using Omniscript RT kit and random primers. The product was diluted to a volume of 150 µl and 5 µl aliquots were used as templates for amplification using the SYBR Green PCR amplification reagent (Qiagen) and semaphorin 3A primers. Amplification was normalized to actin expression in same samples.

### Patient Population

This is a post-hoc study using urine samples collected from a previously published study [8]. Urine samples were prospectively obtained from consenting patients who underwent cardiac surgery using CPB at Cincinnati Children's Hospital for the correction or palliation of congenital cardiac defects during the period of July 2006 to June 2007. Exclusion criteria included preexisting renal insufficiency, diabetes mellitus, concomitant nephrotoxic drug use, and incomplete urine collections. Subjects were enrolled only if their preoperative kidney function was normal, on the basis of an estimated creatinine clearance of >100 ml/min per 1.73 m^2^ as calculated using the Schwartz formula as described previously [31]. To minimize postoperative volume depletion and prerenal azotemia, all subjects received at least 80% of their maintenance fluid requirements during the first 24 hours after surgery and 100% maintenance subsequently. AKI was defined as a 50% increase in serum creatinine from baseline, which occurred, on average, 48 hours after surgery. In addition, each patient with AKI was classified according to the recently described pRIFLE criteria, which is a modification of the RIFLE criteria for use in children [32]. For each patient, six urine samples were obtained that corresponded to times 0, 2, 6, 12, 24, and 48 hours after initiation of CPB. Urine samples were centrifuged at 2000×*g* for 5 minutes, and the supernatants stored in aliquots at −80°C. The research protocol for collection and analysis of these samples was approved by the Cincinnati Children's Hospital Institutional Review Board and Georgia Health Sciences University.

### Semaphorin 3A Quantification by Enzyme-Linked Immunosorbent Assay

Deidentified samples were blinded for semaphorin 3A analysis. Fifty microliters of urine was used for the semaphorin 3A assay. The assay was done using an enzyme-linked immunosorbent assay kit (Catalogue no. MBS732622; My Biosource, CA, USA). Briefly, semaphorin 3A standard, samples and secondary antibody-HRP conjugate were added to antibody-coated 96-well plates and incubated for 1 hour at 37°C. Plates were then washed and color was developed using tetramethylbenzidine substrate, and reaction was arrested by adding sulfuric acid. The color change was measured using a plate reader (Labsystems) at a wavelength of 450 nm. The concentration of semaphorin 3A in the samples was determined by comparing the OD of the samples to the standard curve, with a minimal limit of detection of 10 pg/ml. All measurements were made in duplicate. Urinary semaphorin 3A concentration was expressed as picograms per milligram of urine creatinine. The interassay coefficient of variation for urinary semaphorin 3A was 6.8%.

### Statistical Analyses

SAS version 9.3 was used for all analyses (SAS Institute, Cary, NC), and a significance level of 0.05 was used throughout, controlling for multiple comparisons where necessary. Demographics and clinical outcomes were compared between patients who developed AKI and patients who did not. Continuous variables were compared using the two-sample *t* test, and categorical variables were compared using Fisher's exact test. Estimates of mean values of serum creatinine and urinary semaphorin by group at various time points were calculated using repeated-measures ANOVA, which accounts for correlations of measurements from the same individuals across time. Least square (LS) means and their standard errors (SEMs) are reported. Due to the extreme skewness in the urinary semaphorin values, rank-based repeated-measures analysis was used.

Spearman correlation coefficients were used to show the correlation between urinary semaphorin concentrations at various time points (baseline and at 2, 6, 12, 24, and 48 hours after surgery) and the following clinical outcomes: percent change in serum creatinine, CPB time, hospital length of stay after surgery, and days of AKI.

To measure the sensitivity and specificity for urinary semaphorin, a conventional receiver-operating characteristic (ROC) curve was generated for urinary semaphorin at 2, 6, and 12 hours after the initiation of CPB. We calculated the area under the curve (AUC) to ascertain the utility of semaphorin as a biomarker. An area of 0.5 is expected by chance, whereas a value of 1.0 signifies a perfect biomarker. The optimal urinary semaphorin time point was selected to maximize prediction at the earliest time possible, thus weighing the AUC, timing of measurement, and *P* value from the predictive logistic model. We then identified the values of urinary semaphorin that provided 95% sensitivity, 95% specificity, and optimal sensitivity and specificity using the ROC curve at the best time point.

Univariable and multivariable logistic regression analyses were then undertaken to assess predictors of AKI. Potential independent predictor variables included urinary semaphorin concentration at the best time point, age, sex, race, CPB time, previous heart surgery, and hospital length of stay. Variables were retained in the final model if *P*≤0.05.

## Results

### Immunolocalization of Semaphorin 3A kidney from Normal Animals and Animal which were Subjected to Acute and Chronic Kidney Injury

**Figure 1:**
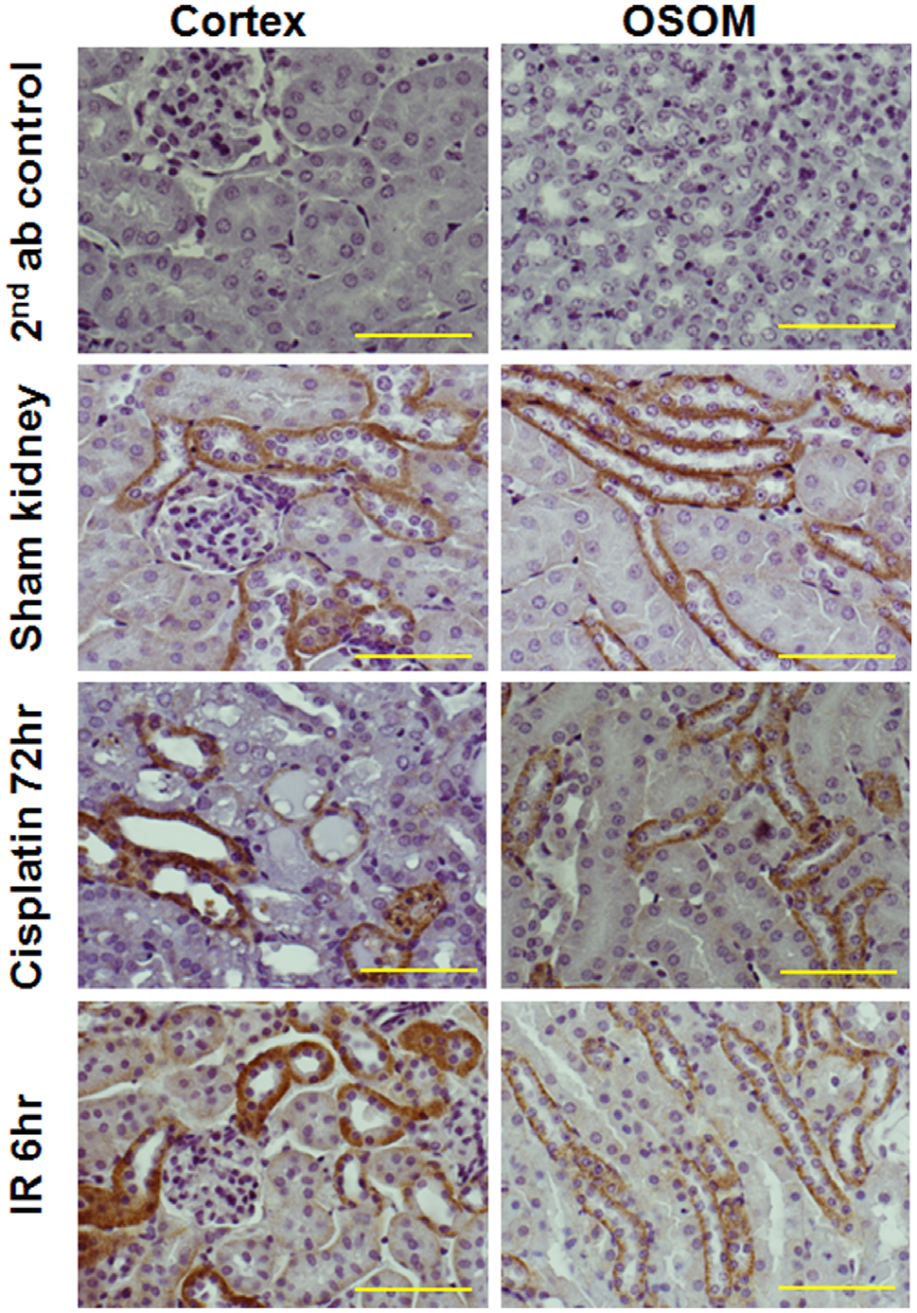
Immunohistochemical localization of semaphorin 3A in kidney after different treatments. Immunohistochemical localization of semaphorin 3A was carried out as described in Materials and Methods. Immunostaining for semaphorin 3A is seen in thick ascending limb of the loop of Henle and distal tubular epithelial cells. Scale Bar: 100 µM.

Immunohistochemical examination revealed that semaphorin 3A was intensely stained in distal tubular epithelial cells and collecting tubules in normal and injured kidney. The intensity of staining is increased after induction of AKI (Figure 1). The identity of collecting tubules is not confirmed. It could be thick ascending limb of the loop of Henle as well.

#### Semaphorin 3A is an early biomarker of ischemic acute kidney injury in experimental animal models

**Figure 2:**
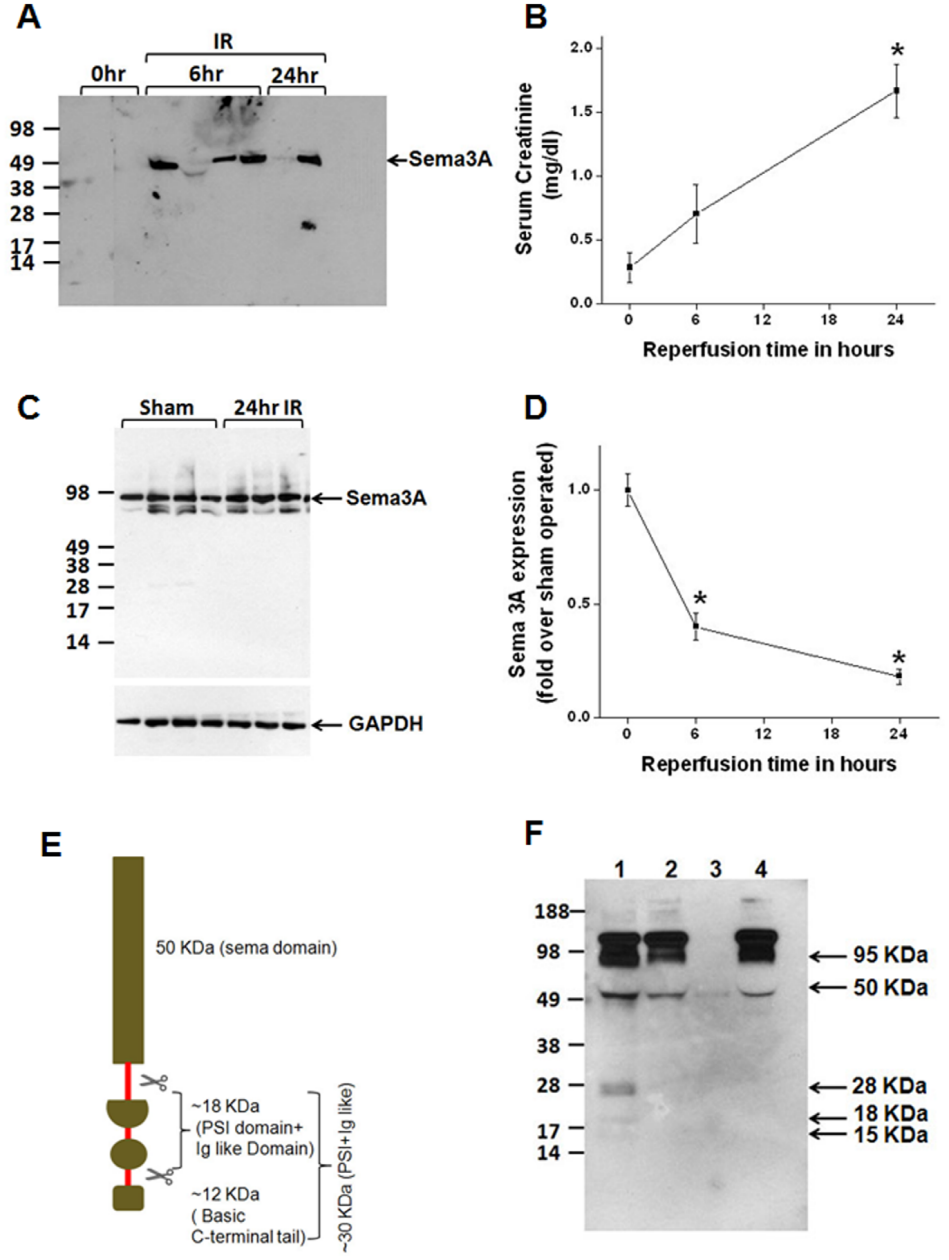
Regulation of semaphorin 3A expression and excretion after ischemia reperfusion injury of the kidney. A. Western blot analysis of semaphorin 3A excretion in mice urine before (0 hr) and at different time point after reperfusion. A large increase was detected at 6 hr and 24 hr after reperfusion. B. Serum creatinine levels before and different time after ischemia reperfusion of the kidney. *p<0.001 vs. 0 hr. C. Semaphorin 3A expression in kidney tissue was analyzed by Western Blot. A single 95 KDa band was observed and increased expression seen at 24 hr. D. RT-PCR analysis of semaphorin 3A expression in the kidney after ischemia reperfusion. Semaphorin 3A mRNA expression is downregulated at 6 and 24 hr after reperfusion of the kidney. *p<0.001 vs. sham operated. E. Graphic representation of known protease (Furin like) cleavage site and expected band of semaphorin 3A. F. Proteolytic cleavage of semaphorin 3A *in vitro*. 250 ng of recombinant semaphorin 3A-Fc chimera was incubated with 10 µl of human urine for 1 hr at 37°C (lane 1), in the presence of 20 mM EDTA (lane 2), human urine alone (lane 3) and recombinant semaphorin 3A alone. Proteolytic release of smaller fragments of semaphorin 3A was inhibited with addition of EDTA. Values are mean ± SEM. n = 3–5.

To determine whether semaphorin 3A excretion is enhanced after ischemia followed by reperfusion, Western analysis was carried out. As shown in figure 2A, semaphorin 3A is undetectable in normal urine whereas a large increase was seen in the first urine collected at 6 hr after reperfusion. This increase persisted even at 24 hr after reperfusion. In contrast, serum creatinine level was significantly elevated only after 24 hr of reperfusion (Figure 2B). To determine whether kidney tissue levels also increased after reperfusion, semaphorin 3A expression was determined by western blot analysis. A modest increase was seen at 24 hr (Figure 2C). In contrast to protein expression, the mRNA expression was down regulated within 6 hr after reperfusion and the downregulation was persisted even at 24 hr. Semaphorin is known to be cleaved by furin like proteases to release multiple fragments (Figure 2E) [33]. Since urine semaphorin 3A molecular mass is 50 KDa, we analyzed whether proteases from urine generate 50 KDa and lower fragments. Incubation of recombinant semaphorin 3A Fc chimera with human urine for 1 hr released 50, 28, 18 and 15 KDa lower sized fragments which match the predicted molecular weight of proteolytic fragment of semaphorin 3A. In addition, release of mature 95 KDa protein (which includes the signal peptide) also increased from the Fc chimera. Interestingly, addition of EDTA completely blocked the proteolytic cleavage of semaphorin 3A suggesting that metalloproteases may mediate this release of lower size fragments in urine.

**Figure 3:**
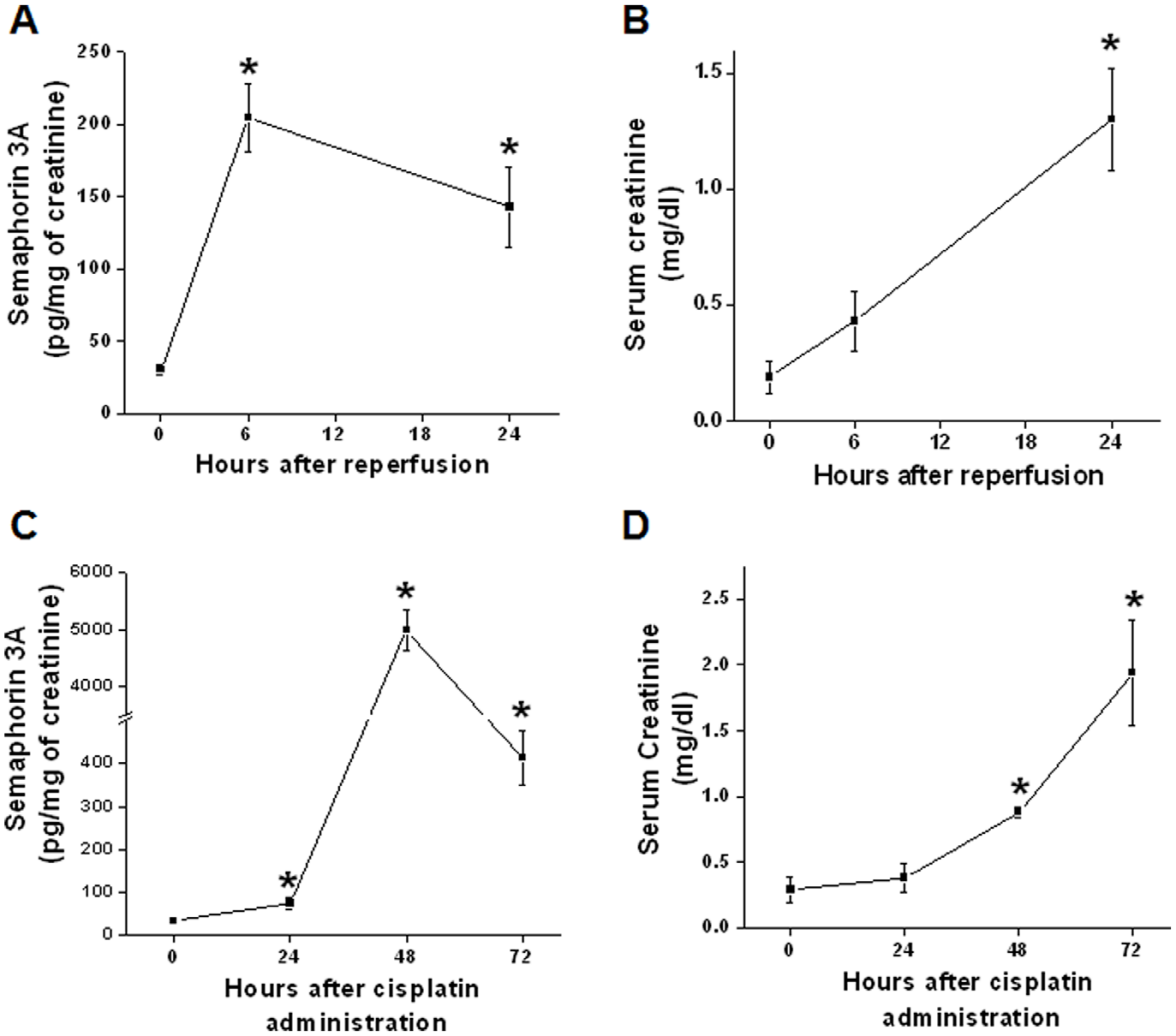
Quantification of urine semaphorin 3A in different forms AKI and diabetes in mouse. Semaphorin 3A was quantified using ELISA kit as described in Methods. A. Semaphorin 3A in urine from animals subjected to sham surgery (0 hr) and different time after reperfusion. Ischemia reperfusion rapidly increased urinary excretion of semaphorin 3A. *p<0.005 vs. 0 hr. B. Serum creatinine at different time after reperfusion. *p<0.001 vs. 0 hr. C. Semaphorin 3A excretion in urine before and different time after cisplatin administration. Cisplatin administration significantly increased the excretion of semaphorin 3A at 24, 48 and 72 hr. *p<0.005 vs. 0 hr. D. Serum creatinine at different time after administration of cisplatin. *p<0.05. Values are mean ± SEM. n = 6–8.

To determine absolute quantity of semaphorin 3A in mouse urine and serum after AKI, we had quantified using an ELISA kit. Sema3A is significantly increased at 6 ad 24 hr after reperfusion whereas serum creatinine is increased significantly only at 24 hr after reperfusion (Figure 3A and B). This confirms the above observation with western blot analysis that semaphorin 3A is an early diagnostic biomarker of AKI. Similarly, semaphorin 3A is also increased significantly at 24, 48 and 72 hr after cisplatin administration where serum creatinine rise was seen 72 hr after cisplatin administration (Figure 3C and D).

**Figure 4:**
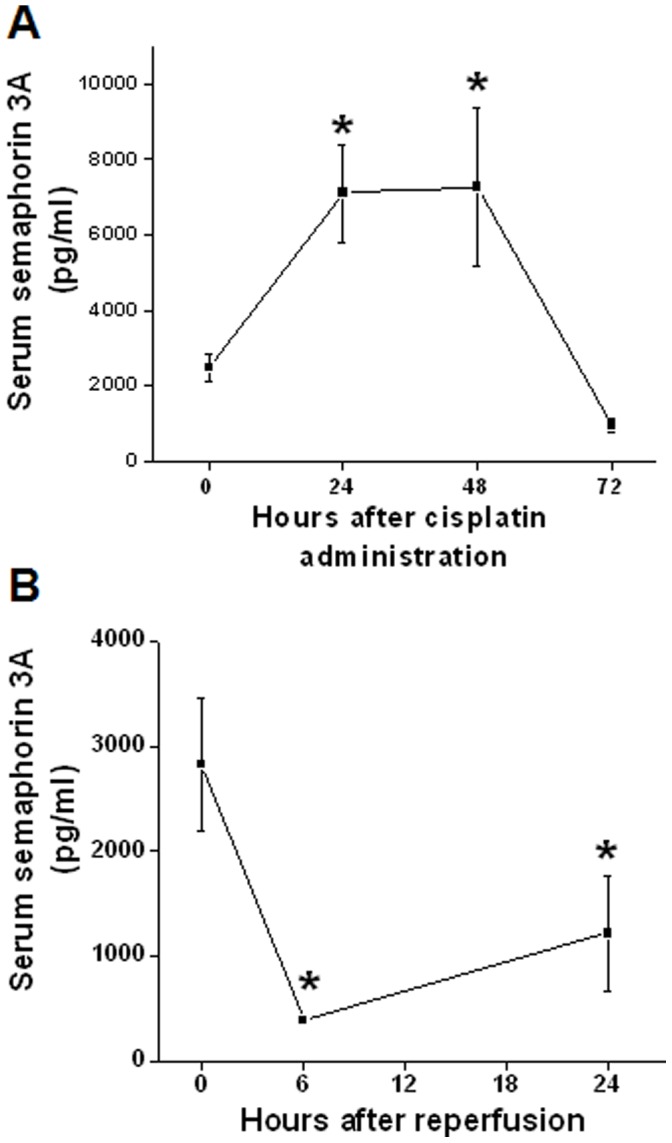
Quantification of serum semaphorin 3A in different forms AKI and diabetes in mouse. Semaphorin 3A was quantified using ELISA kit as described in Methods. A. Circulating levels of semaphorin 3A before and different time after cisplatin administration. Cisplatin administration was significantly upregulated semaphorin 3A in the blood. *p<0.005 vs. 0 hr. B. Circulating levels of semaphorin 3A in sham operated (0 hr) and different time after reperfusion. Ischemia reperfusion rapidly downregulated circulating semaphorin 3A. *p<0.005 vs. 0 hr. Values are mean ± SEM. n = 4–6.

Unlike urine semaphorin 3A, circulating semaphorin 3A is not increased after reperfusion but rather rapidly downregulated (Figure 4B). In contrast to ischemia reperfusion, cisplatin administration significantly upregulated at 24 and 48 hr (Figure 4A) suggesting differential regulation of circulating semaphorin 3A in response to hypoxia and toxic kidney injury. Interestingly circulating semaphorin 3A is also upregulated after induction of diabetes (Figure 4C).

### Patient Characteristics and Renal Function Changes

During the period of enrollment, 120 subjects underwent CPB at our institution. Of these, 60 subjects met the inclusion criteria for this study. The most common reason for excluding a subject was incomplete urine collections. AKI occurred in 26 children (43%) within a 3-day period. No significant differences were noted between the two groups with respect to age, race, need for dialysis, or mortality (Table 1). Children who developed AKI had significantly longer CPB times compared with those who did not develop AKI (*p*<0.0001), and also experienced significantly longer hospital stays (*P = *0.0006). Figure 1 shows the changes of serum creatinine concentrations after CPB for children who developed AKI and those who did not. During the first 24 hours after CPB, serum creatinine did not differ significantly between the two groups. Significant differences between groups were seen by 48 hours after surgery and were maintained until 5 days after surgery.

**Table 1:** Descriptive statistics of patient characteristics.

Parameter	AKI	No AKI	*P*
*n*	26	34	–
Age, yr	4.3±4.5	4.0±4.6	0.81^a^
Male, %	35	65	0.04^b^
White, %	81	91	0.28^b^
Prior surgery, %	38	35	1.0^b^
Bypass time, min	188.4±62.6	91.4±47.8	<0.0001^a^
Creatinine change, %	171.3±133.9	11.6±12.2	<0.0001^a^
Duration of AKI, d	4.8±4.5	–	–
Hospital stay, d	13.8±11.6	4.8±2.9	0.0006^a^
Dialysis, %	8	0	0.18^b^
Death, %	4	0	0.43^b^

Means ± standard deviation (SD) are reported for continuous measures, percentages are reported for categorical variables.

a Welch modified two-sample *t* test.

b Fisher exact test.

### Associations of Semaphorin 3A with Patient Characteristics

Semaphorin was negatively associated with patient age at baseline and at 24 hours (Table 2). Lower semaphorin levels before surgery were predictive of percent change in serum creatinine postsurgery and duration of AKI. Higher semaphorin levels at all time points between 2 and 12 hours were significantly associated with greater percent change in serum creatinine, longer CPB time, longer hospital length of stay, and longer duration of AKI. Higher semaphorin levels at 24 hours were significantly associated with longer hospital length of stay. Semaphorin levels at 48 hours were not significantly associated with any post-surgery outcomes.

**Table 2:** Spearman correlation coefficients of semaphorin 3A with clinical characteristics.

	Age	Percent Change in Serum Creatinine	CPB Time	Hospital Length of Stay	Days AKI
Baseline	−0.27^a^	−0.26^a^	−0.16	−0.13	−0.26^a^
2 h	0.10	0.50^b^	0.48^b^	0.43^b^	0.59^b^
6 h	0.03	0.42^b^	0.44^b^	0.49^b^	0.49^b^
12 h	−0.09	0.43^b^	0.44^b^	0.53^b^	0.39^b^
24 h	−0.32^a^	0.17	0.13	0.40^b^	0.21
48 h	−0.24	0.08	0.10	0.23	0.11

a *P*≤0.05.

b *P*≤0.002.

### Urinary Semaphorin 3A Predicts AKI after Cardiac Surgery

**Figure 5:**
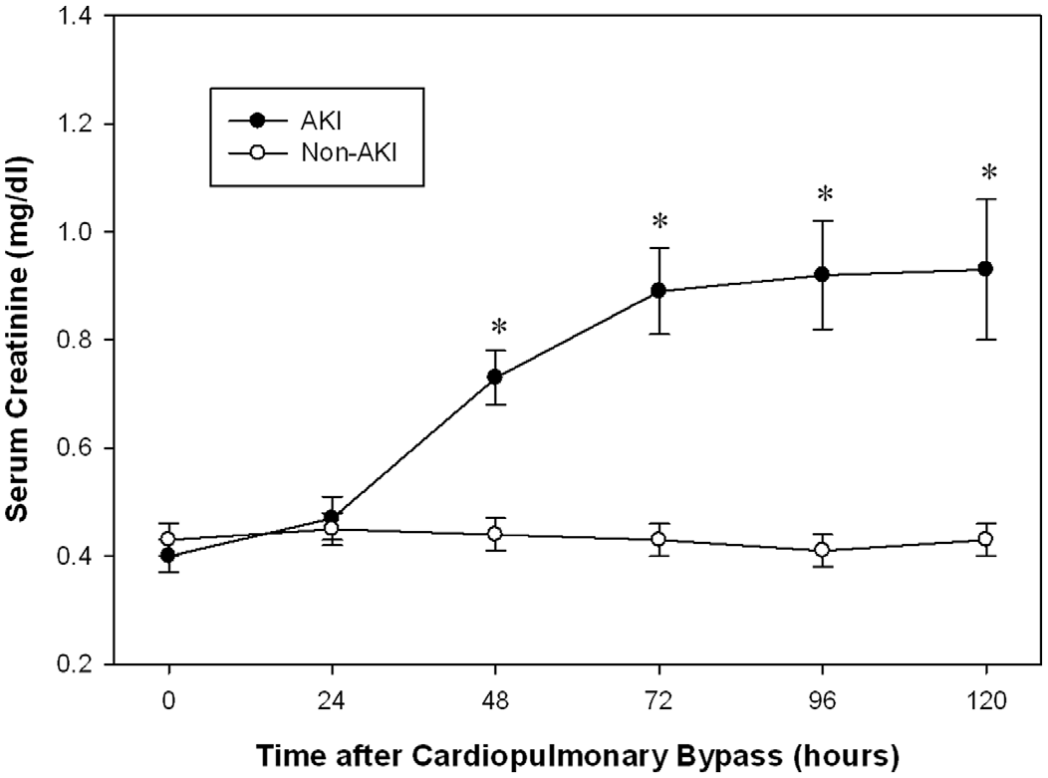
Changes in serum creatinine (LS mean±SE) at various time points after cardiac surgery in the non-AKI and AKI group. *p≤0.0002 for differences between groups by repeated measures ANOVA.

**Figure 6:**
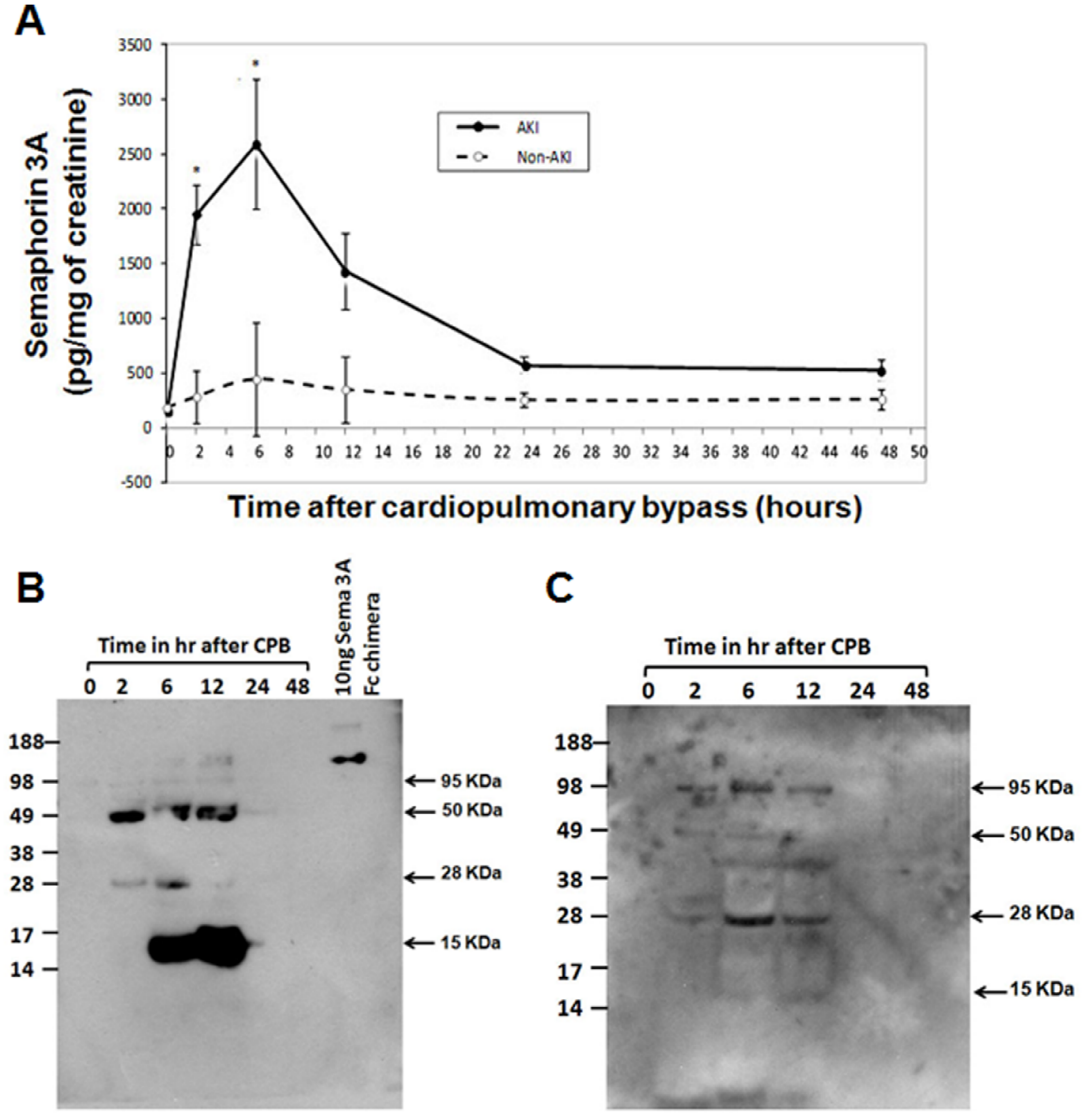
Quantification of semaphorin 3A in urine from patients who were undergone cardiopulmonary bypass surgery (CPB). A. Changes in urinary semaphorin concentrations at various time points after CPB surgery in non-AKI and AKI patients. Error bars are LS mean ± SEM. *p≤0.005 for differences between groups (non-AKI and AKI) by repeated-measures ANOVA. B. Western blot analysis of urine samples from a patient with AKI. Blot was probed with rabbit polyclonal antibody from Abcam. C. Western blot analysis of urine samples from a patient with AKI. Blot was probed with goat polyclonal antibody from Santacruz Biotechnology.

Currently used diagnostic biomarker serum creatinine start to rise significantly at 48 hr after initiation of CPB and remain elevated after that in patient categorized as AKI (Figure 5). In contrast, urinary semaphorin is increased significantly in patients who developed AKI by 2 hours after the initiation of CPB, peaked at 6 hours after surgery, and was no longer significantly elevated 12 hours after surgery (Figure 6A). Patients who did not develop AKI experienced a much smaller increase shortly after surgery that resolved to baseline by 12 hours after surgery. ELISA results were confirmed by western blot analysis using two different polyclonal antibodies raised in two different species against semaphorin 3A (Figure 6B and C).

**Figure 7:**
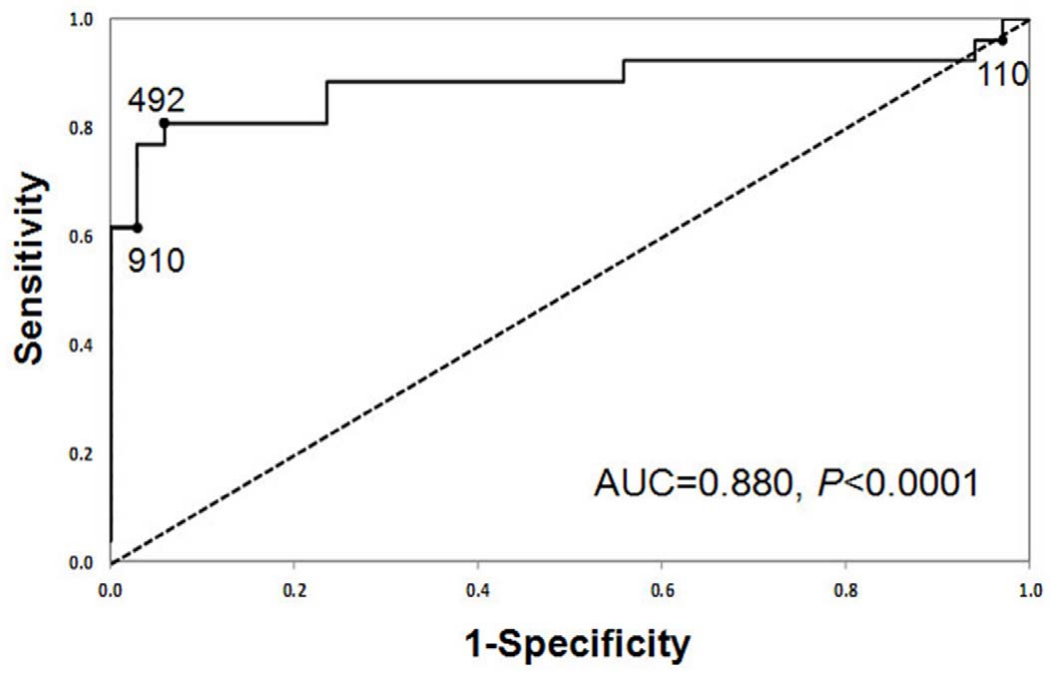
ROC curve analysis for urinary semaphorin at 2 hours after cardiac surgery. The values 109.8, 492.1, and 910.0 are urinary semaphorin concentrations (in picograms per milligram urine creatinine) at 2 hours after CPB, which correspond to 96% sensitivity, optimal sensitivity and specificity, and 97% specificity, respectively.

Conventional ROC curves for AKI *versus* no AKI were generated for urinary semaphorin at 2, 6, and 12 hours after surgery. The AUCs of the three ROC curves are 0.880 (*p*<0.0001), 0.810 (*p*<0.0001), and 0.737 (*p = *0.0009), respectively. After weighing the AUC, timing of measurement, and *P* value from the predictive logistic model, the optimal urinary semaphorin time point was selected at 2 hours after surgery. Figure 7 displays the unadjusted ROC curve for urinary semaphorin at 2 hours after cardiac surgery. The sensitivities and specificities for three semaphorin concentrations obtained at the 2-hour time point are listed in Table 3, corresponding to 95% sensitivity, optimal sensitivity and specificity, and 95% specificity. A cutoff value of 492.1 pg semaphorin per mg of urinary creatinine at 2 hours after cardiac surgery yields the optimal combination of sensitivity (81%) and specificity (94%).

**Table 3:** Urinary semaphorin 3A test characteristics at different cutoff values.

Cutoff Value for Semaphorin 3A,pg/mg of Urine Creatinine	Sensitivity	Specificity	Positive Predictive Value	Negative Predictive Value
110	0.96	0.03	0.43	0.50
492	0.81	0.94	0.91	0.87
910	0.62	0.97	0.94	0.77

The cutoff values are urinary semaphorin concentrations at 2 h after cardiopulmonary bypass, which correspond to 95% sensitivity, optimal sensitivity and specificity, and 95% specificity, respectively.

**Figure 8:**
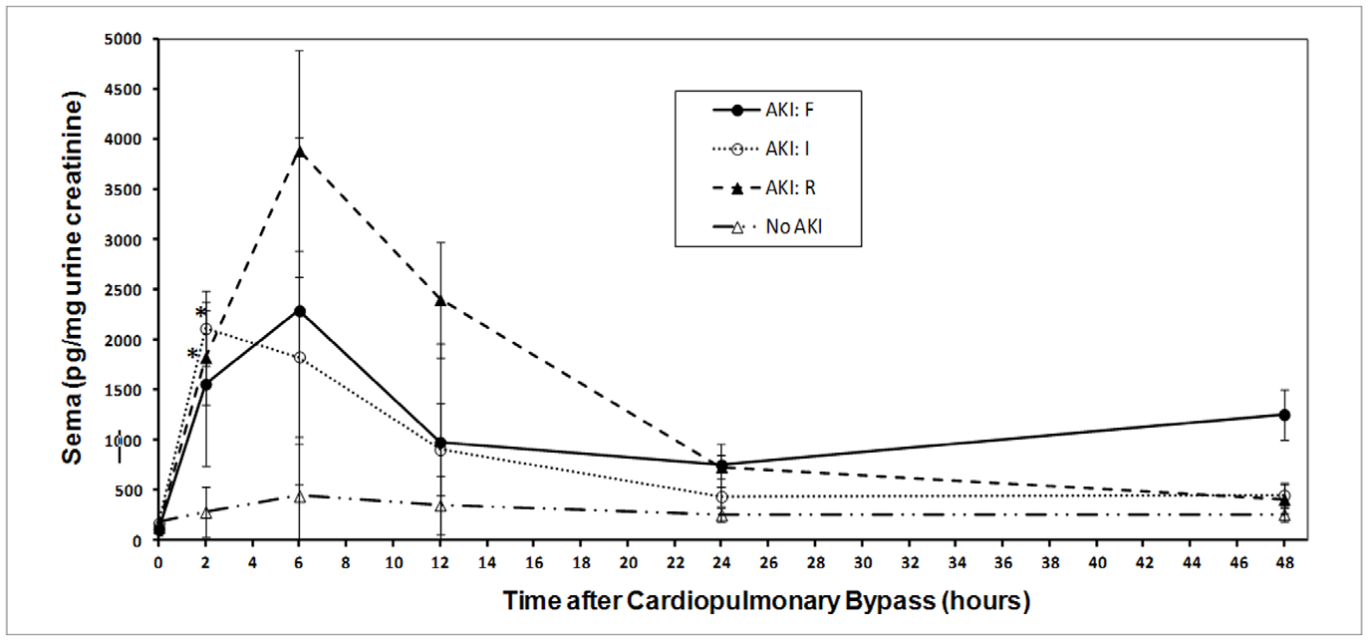
Changes in urinary semaphorin 3A concentrations at various time points after CPB surgery in non-AKI and AKI patients, stratified by pRIFLE categories. *p≤0.004 for differences between groups (non-AKI and each of the pRIFLE categories) by repeated-measures ANOVA.

During the past decade, two major classification systems for AKI have emerged (RIFLE and AKIN), based on serum creatinine and urine output criteria. A modification of the RIFLE criteria was suggested for pediatric use (pRIFLE), substituting serum creatinine values with estimated creatinine clearance. Several recent pediatric AKI studies have employed the pRIFLE criteria to report on AKI incidence and severity [32]; [34]. Among the 26 subjects who developed AKI, nine were classified as being in the risk (R) category, 14 in the injury (I) category, and three in the failure (F) category, on the basis of pRIFLE criteria. Analysis of semaphorin concentrations by pRIFLE classification revealed that both the risk group and the injury group differed from no AKI at 2 hours (all *p*<0.004; Figure 8). No other significant differences were found among the pRIFLE groups.

Univariable logistic regression identified longer CPB time (*p*<0.0001), male gender (*p = *0.0230), longer hospital length of stay (*p = *0.0007), and higher semaphorin concentrations at 2 hours (*p* = 0.0015), 6 hours (*p = *0.0025), 12 hours (*p = *0.0038), and 24 hours (*p = *0.0086) as significantly associated with higher odds of AKI. A stepwise logistic regression analysis was used to determine the most parsimonious model given a set of potential variables for predicting AKI. Potential variables for this model included age, sex, CPB time, previous cardiac surgery, hospital length of stay, and semaphorin at the selected optimal time point (*i.e.*, 2 hours after surgery). The final model revealed that sex, CPB time and semaphorin concentration at 2 hours after surgery were the only significant independent predictors of AKI in our cohort. The estimated odds ratio for every 100-pg/mg urinary creatinine increase of semaphorin at 2 hours after surgery was 2.191 (95% confidence interval: 1.001 to 4.502; *P = *0.0328). The estimated OR for every 1-min increase of CPB time was 1.049 (95% CI: 1.012 to 1.087; *P = *0.0087), and the estimated OR for male gender was 0.018 (95% CI: 0.001 to 0.529; *P = *0.0200).

## Discussion

This is the first study to demonstrate that urinary excretion of semaphorin 3A is an early predictive biomarker of human AKI. In pediatric patients undergoing CPB, subjects that developed AKI displayed significantly increased urinary semaphorin 3A levels within the first 2 h of the initiation of CPB, preceding the rise in serum creatinine by 48–72 hours (Figure 5).

In preclinical studies of mouse models, semaphorin 3A is not expressed in proximal tubular epithelial cells and glomerular messangial cells but highly expressed in the distal tubular and collecting duct epithelial cells. Moreover, it is undetectable in normal urine. However, semaphorin excretion is increased within few hours after reperfusion and can be easily detected in the urine. These observations suggest that semaphorin 3A is an early marker of acute kidney injury. However, the mechanism of enhanced excretion following reperfusion is not clear.

The role of semaphorin 3A in kidney pathophysiology is unknown. Our unpublished studies showed administration of semaphorin 3A did not suppress or exaceberate ischemia reperfusion injury. Semaphorin 3A is a known anti-angiogenic molecule but whether it regulates angiogenesis is unknown. In addition semaphorin 3A is known to regulate cell migration and adhesion. Since epithelial cell proliferation and migration is known to occur immediately after acute injury, it is possible that semaphorin may involve in these processes. However, this possibility may not pertain to proximal tubular epithelial cells as semaphorin 3A is not expressed in these cells.

Our results indicate that semaphorin 3A is a powerful early biomarker of AKI that precedes the increase in serum creatinine in experimental animal models by a day. The magnitude of rise supports the notion that semaphorin 3A represents a useful early diagnostic biomarker of acute kidney injury. Consistent with the experimental animal model, human studies also indicate semaphorin 3A is a highly discriminatory biomarker with a wide dynamic range and cutoff values that allow for risk stratification. Indeed, we found that other variables such as patient demographics and previous cardiac surgery were not predictive of AKI, and could not be used for risk assessment in our cohort. However, early urinary semaphorin 3A levels were associated with important clinical outcomes such as severity of AKI, days of AKI and length of hospital stay.

We have not measured serum semaphorin 3A levels in this cohort of patients to determine whether semaphorin 3A level in serum also has a predictive value for AKI. Due to its size and distal tubular expression pattern, urinary semaphorin 3A is unlikely to be derived from glomerular filtration. These considerations suggest that the early increase in urine semaphorin 3A in subjects destined for AKI is a direct reflection of kidney tubule injury.

Both animal and human urine semaphorin 3A proteins appear as multiple proteolytic fragments apart from full length protein. It is known from the literature that semaphorin 3D is cleaved by furin like proteases into multiple smaller fragments [33]. We observed similar degradation pattern of semaphorin 3A suggesting a similar proteolytic processing is involved for semaphorin 3A as well. The predicted molecular weight of fragment matches with observed band in urine. Moreover, *in vitro* studies show that incubation of human urine with recombinant protein also releases a similar protein fragments. These results confirm identity of protein fragments detected in urine is indeed derived from semaphorin 3A. The importance of proteolytic process in the kidney pathophysiological process is not clear. Whether smaller fragments or fragment of semaphorin 3A are more efficient in binding receptor is unknown. However, proteolytic processing is required to generate an active full length protein (98 KDa) for semaphorin 3D. Moreover, further processing by furin like endoprotease completely abolished its repulsive activity on the neurons [33]. Therefore, it is possible that proteolytic clevage of semaphorin 3A into smaller fragment in urine will inactivate its activity.

Our study has strengths. First, we have identified a new biomarker for acute kidney injury and validated it in human samples. Second, we prospectively recruited a relatively homogeneous cohort of pediatric subjects in whom the only obvious etiology for AKI would be the result of cardiac surgery. Third, all subjects started with normal kidney function and low levels of semaphorin 3A in the urine. The study design allowed for the temporal definition of altered semaphorin 3A concentrations following cardiac surgery, and a direct comparison with changes in serum creatinine, the current gold standard for the definition of AKI. Fourth, we adjusted for changes in urine concentration by correcting urinary semaphorin 3A concentrations with urinary creatinine. Most of all, we also carried out a similar study but in much more complex ICU patient populations showing a high degree of sensitivity and specificity (see accompanying paper). Our results also support that semaphorin 3A is a very useful predictive early biomarker irrespective of the patient population.

The current study also has limitations. It is a single-center pilot study of pediatric subjects with congenital heart defects undergoing elective cardiac surgery. Thus, these results will need to be validated in a larger population, including adults with the usual confounding variables and comorbid conditions that normally accumulate with increasing age. Recent studies have uncovered other AKI biomarkers such as neutrophil gelatinase-associated lipocalin [8]; [35]–[37], interleukin-18 [9], liver-type fatty acid binding protein [38]; [39], and kidney injury molecule-1 [40] in clinical cohorts similar to that employed in this study. However, all biomarkers have individual strengths and weaknesses. Given the multifactorial etiologies of AKI (34), it appears unlikely that any single biomarker will suffice. Indeed, even in the cardiac surgical population, measurements of single urinary biomarkers such as neutrophil gelatinase-associated lipocalin have yielded a wide range of AUCs (0.59–0.99) for the prediction of AKI [41].

It is anticipated that a collection of strategically selected candidates, including semaphorin 3A as reported herein, may prove of value for early and rapid diagnosis of AKI and its clinical outcomes. This approach has been recently proposed to improve the early diagnosis and risk stratification in cardiovascular diseases [42]; [43]. It is also likely that emerging AKI biomarker panels will enable the timely initiation of interventions such as atrial natriuretic peptide [44] and insulin-like growth factor [45] that have been successful in smaller, phase II-level efficacy studies but not in larger phase III trials. Possibly, these interventions will be successful if they are initiated at the onset of AKI (as determined by predictive biomarkers) rather than waiting several days for serum creatinine to rise. In addition, animal studies have identified, and continue to reveal, novel therapies such as growth factors, stem cell therapies, anti-apoptotic, anti-inflammatory, and anti-oxidant approaches that are effective in early AKI, prior to the rise in serum creatinine [6]. It is also likely that emerging biomarker panels will enable these promising agents to be investigated in humans with AKI, especially in temporally defined clinical situations such as subjects undergoing CPB.
